# Decoupling evolution of water footprint of energy and its driving factors: Evidence from Northeast China

**DOI:** 10.1371/journal.pone.0317031

**Published:** 2025-01-14

**Authors:** Liying Cui, Shaoping Li, Hengshuo Zhang

**Affiliations:** School of Economics and Management, Northeastern Petroleum University, Daqing, China; Bina Nusantara University, INDONESIA

## Abstract

Energy and water are interlinked and inseparable resources of vital importance to the survival and development of human society. Exploring the relationship between energy and water is of great practical significance for the sustainable development of resources. The uneven regional distribution of energy and water in China has exacerbated energy-related water shortages. Base on the water footprint of energy (WFE), this paper takes Northeast China, the old industrial base, as the research object, and explores the regional distribution characteristics and development trend of WFE, so as to provide quantitative basis for the development of regional energy transformation in coordination with energy and water. This study uses the ISO model and the decoupling model, and analyzes the spatial and temporal changes in the water footprint (WF) of energies and the decoupling effects in Northeast China from the perspective of energy production. The findings show: (1) the WFE grew slowly, and the WF of power gradually exceeded that of fossil energy; (2) the spatial distribution of WFE was unbalanced, with higher WF of fossil energy in Heilongjiang and higher WF of power in Jilin and Liaoning; (3) the decoupling status of WFE from GDP was better than that of WFE from total water consumption. This study also analyzes the driving factors of indicators of water endowment, economic development, energy consumption, environmental governance and life quality, on the WFE based on the Lasso model, and provide policy implications for the coordinated development of energy and water in Northeast China.

## 1 Introduction

The world sustainable development goals (SDGs) issued by the United Nations emphasize that the optimal adaptation of water and energy resources has an essential contribution to achieving sustainable development in all world countries [1, 2]. Water and energy are interdependent and inseparable [3]. Energy production, processing, and transport processes require large amounts of water [4, 5]. Approximately 90% of the world’s energy production is water-intensive, and 15% of the world’s water resources are supplied for energy production [6]. In the context of rapid global population growth, nearly 900 million people suffer from water scarcity [7], and water supply has become an essential issue for developing the energy sector [8]. In addition, with rapid social and economic development, the global demand for energy is expected to increase by 30% by 2040 [9]. The need for water will increase by 20–30% by 2050 [10, 11], and freshwater has been identified as a significant risk to humanity in the coming decades [12, 13]. Therefore, in-depth analyses of water demand in the energy production process, the optimization of energy facilities such as hydropower with the help of research in technical fields (such as fluid dynamics) [14], and the minimization of water consumption are essential to ensure the sustainable development of energy and water.

China, as the world’s largest energy producer and consumer, grew its energy production by 2.5% in 2020, and accounts for 26.11% of global primary energy consumption [15]. In the process of globalization, as a developing country with more advanced industrialization, China’s energy model relies on fossil fuels such as coal and oil, and its economic growth is slightly decoupled carbon emissions from energy consumption [16]. The energy industry was a vital water consumer in China, which accounted for 6.6% of total water consumption in 2014 [17]. Most of China’s energy bases are located in the water-scarce northern region, where energy resources are abundant. Energy investment is an important support for local economic growth, which leads to a large amount of water demand, so the development of energy industry does not match with the water resource management policies of the regions [18]. It can be seen that the continuous growth of energy production and the shortage of available water make the "energy-water crisis" increasingly prominent [19]. As an old industrial base in China, the irrational industrial structure and over consumption of water in Northeast China are apparent [20], which has become the region’s main bottleneck for economic revitalization and development. Therefore, realizing the efficient use of energy and water is of strategic importance to the sustainable development of the economy of Northeast China.

There is a complex relationship between energy and water as raw materials for each other in the production process [21]. In the recent years, researches on the relationship between energy and water have been relatively abundant, with studies focusing on water consumption in the process of energy production and consumption [22–24], energy consumption in the process of water production [25–27], measurement of two-way consumption of energy and water [28–30], the evaluation of energy-water policies and the analysis of development strategies [31, 32], the systematic linkage of water-energy-food and the competitive relationship of resources [33, 34], and spatial distribution characteristics and coordinated development of carbon-energy-water footprint [35, 36]. For example, Okadera et al. [37] evaluated the WF of energy production in Liaoning Province, China, from the perspective of consumption. Jinyoung and Hana [38] estimated the energy intensity of each stage of the water cycle for 17 cities in South Korea from 2012 to 2017. Gu et al. [31] estimated the water-saving results of the implementation of energy-saving policies in the ferrous metal industry, non-ferrous metal industry, petrochemical industry, construction industry, electric power industry, and critical light industry from 2005 to 2010. Ghani et al. [33] presented the relationship between water, energy, and food and its environmental impact on bioethanol in Pakistan.

As important resources for human society, the coordinated development of energy and water with the economy is an intrinsic requirement for promoting regional ecological protection and achieving high-quality development [39, 40]. Existing studies on the coordinated development of energy, water, and economy were quite rich, mainly centered on the dimensions of countries and regions [41–43], provinces and cities [44–46], and industries and sectors [47, 48]. Zhang et al. [45] analyzed the spatial relationship between the WF of energy of the Jixian Bend of the Yellow River and water from 2000 to 2019 using the standard deviation ellipse model and the WF stress index. Wang et al. [46] used the decoupling model to analyze the decoupling relationship between water footprint and GDP in Sichuan Province, and the result was a weak decoupling state with relatively coordinated development. Liu et al. [48] assessed the dependence of water-energy-economy of China’s iron and steel industry in the period of 2013–2019. Existing studies have paid less attention to the synergistic relationship between the WF of energy and water consumption, the WF of energy and economic development. Energy production consumes a high proportion of water in China, so an in-depth analysis of the relationships based on the perspective of Northeast China is of great significance in achieving the strategic goals of sustainable growth of energy, water and economy in Northeast China.

In addition, the analysis of driving factors of WFE, as a research hotspot in the period of sustainable development of resources and economy, and the methodology adopted by existing scholars to study the driving factors of WFE also varied. For example, Yan et al. [49] revealed the driving effects of water consumption in the Yangtze River Delta region as intensity effect, scale effect, and linkage effect based on the GDIM model. Zhang et al. [50] used the EE-MRIO model to analyze five aspects of water withdrawal intensity, production structure, energy demand structure, per capita energy demand, andpopulation drivers of inter-provincial energy water withdrawal in China from 2007–2012. Based on the perspective of water-energy-food linkage, Ke et al. [51] selected the LMDI method to analyze the influencing factors of WF by using China’s data from 1999–2019. They found that the production effect is the dominant factor in promoting the growth of China’s WF, whereas water use efficiency is the main factor slowing down the growth of WF in China. Zhang et al. [52] used the Panel Pool Mean Group and autoregressive distributive lag model to analyze the relationship between GDP, population, grain yield, fertilization, urbanization rate, and the agricultural water footprint in the Hexi corridor in China.

In summary, the shortcomings of existing researches on the WFE include: firstly, there are a lot of studies on the measurement of WFE based on multiple categories, but fewer research results on the measurement of WF of clean power (nuclear, wind, and solar); secondly, the research on the relationship of energy, water and economy involves multiple fields, but there are few analyses of the coordination relationship between WFE and water, WFE and economy; thirdly, there are limitations in the existing methods of analyzing the driving factors of WFE. Therefore, the main objective of this study is to analyze the evolutionary characteristics and decoupling effects of the WF of traditional energies and clean power in Northeast China, as well as to explore the heterogeneity of the driving factors of WFE between regions and energies.

Compared with previous studies, the marginal contributions of this paper are: first, measuring the WF of traditional energies and clean power in Northeast China, and analyzing the evolutionary trend of WFE between regions and energies; second, analyzing the decoupling effects of WFE in regions with total water consumption and economic growth, and identifying synergistic relationships between various types of energy and water consumption and economic growth; and third, identifying the driving factors in terms of water endowment, economic development, energy consumption, environmental quality, and life quality, and identifying the driving factors of WFE in different regions and energies.

## 2 Materials and methods

### 2.1 Description of the study areas

Northeast China is an essential old industrial base of China, rich in coal, oil, and other mineral resources. It has made significant historical contributions to forming China’s independent and complete industrial and national economic systems, reform and opening-up, and modernization.

Energy consumption in Northeast China had increased by 122 million tonnes of Standard Coal Equivalent (SCE) from 2005 to 2020, and such a large amount of energy consumption required a large amount of water in the energy production process. The total water resource of Northeast China accounted for only 7% of the country’s water resource, and with the rapid development of social and economic development, the total water consumption in the region was increasing. The contradiction between supply and demand of water was becoming more and more prominent, which restricted the sustainable development of industrial and agricultural production and the economy in the region.

### 2.2 Models

#### 2.2.1 ISO model

The WFE is the total amount of water consumed by energy throughout its production life cycle. According to the correlation between the energy production process and water, the WFE is divided into direct water footprint and indirect water footprint; the direct water footprint refers to the direct consumption of water in the energy production process, and the indirect water footprint refers to the consumption of water from materials or other energies that are inputs in this energy production process. The WFE is also divided into blue water footprint of energy, which refers to the amount of surface runoff and groundwater required for energy production, and the grey water footprint of energy refers to the amount of water required to absorb and assimilate pollutants discharged during energy production. For the measurement of WFE, this paper adopted the energy-water footprint model based on the ISO standard for measurement [53], and the calculation formula was as follows:

$$PWFE_{n}=PWFE_{direct}+PWFE_{indirect}=PWFE_{blue,direct}+PWFE_{grey,direct}+PWFE_{blue,indirect}+PWFE_{grey,indirect}$$ (1)

Where PWFE_n_ represents the WF of per unit energy production for category n, PWFE_direct_ is the meaning of the direct water footprint, PWFE_indirect_ is the indirect water footprint, PWFE_blue,direct_ (PWFE_b,d_) means the direct blue water footprint, PWFE_grey,direct_ (PWFE_g,d_) denotes the direct grey water footprint, PWFE_blue,indirect_ (PWFE_b,in_) is the indirect blue water footprint, PWFE_grey,indirect_ (PWFE_g,in_) is the indirect grey water footprint, and is expressed in billions of cubic metres. PWFE_blue_ is measured according to the water consumption in the direct and indirect production and processing of energy, and PWFE_grey_, is measured using the Water Footprint Network (WFN) model [54], and PWFE_grey,direct_ is measured in the same way as PWFE_grey,indirect_, with the following formula:

$$GWFE=\frac{G}{C_{\max}{‐C}_{\mathrm{nat}}}=\frac{L\times V_{p}}{C_{\max}-C_{nat}}$$ (2)

Where GWFE is the grey water footprint of energy, G represents the emission of pollutants in the energy production process (mg/GJ), L is the emission of wastewater in the energy production process (m^3^/GJ), V_p_ is the amount of pollutant concentration in the discharged wastewater (mg/m^3^), C_max_ represents the maximum concentration of pollutants that can be accepted in the water (counting the primary pollutant in the wastewater, COD), and C_nat_ is the initial concentration of pollutants in the natural water quality.

Based on the calculation of PWFE, the WFE was derived as follows:

$$WFE=\sum_{n=1}^{n}\left(PWFE_{n}\times P_{n}\right)$$ (3)

Where P_n_ is the production of the nth type of energy. The measurement of WFE in the paper involves fossil energy and power, where fossil energy includes coal, crude oil, and natural gas, and power includes thermal, hydropower, and other power (nuclear, wind, and solar).

#### 2.2.2 Decoupling model

The concept of decoupling has been gradually extended to research fields such as environmental economics since its proposal in the 1960s. It is commonly used to describe the relationship between environmental pollution and economic growth, and there are numerous methods for measuring decoupling indicators. The OECD quantified the interrelationship between economic growth and the development of environmental quality by constructing decoupling factors, where the specificity of the initial and terminal value selections could lead to result biases [55]. The decoupling model that constructd the relationship between environmental load and economic growth based on the IGTX equation was susceptible to the influence of other non-environmental factors. The shorter the research period, the more likely the decoupling indicators were to be distorted [56]. The Tapio model [57] used the flexible analysis method based on time scales to reflect the decoupling relationships between variables. It was generally not constrained by the length of the research period and also had a strong ability to identify decoupling states, effectively alleviating the biases caused by the first two models [58].

The Tapio model has a strong capability to identify various decoupling states and is often used to measure the relationship between economic growth and resource environment. In this paper, based on the decoupling theory and the Tapio model, the changes in absolute and relative quantities were combined to construct a decoupling index in terms of the ratio of growth rates [59], which was calculated as shown in Eqs (4)–(5). TWC is the total water consumption, WFE_T_ and WFE_T-1_ are WFE in year T and T-1, TWC_T_ and TWC_T-1_ are TWC in year T and T-1, GDP_T_ and GDP_T-1_ are the gross domestic product in year T and T-1, and e_1_ and e_2_ are the decoupling indices of WFE-TWC and WFE-GDP, respectively, which represent the changes in the relationship between WTC and WFE, GDP and WFE.

$$e_{1}=\frac{\Delta WFE_{t}}{\Delta TWC_{t}}=\frac{(WFE_{T}-WFE_{T-1})/WFE_{T-1}}{(TWC_{T}-TWC_{T-1})/TWC_{T-1}}$$ (4)

$$e_{2}=\frac{\Delta WFE_{t}}{\Delta GDP_{t}}=\frac{(WFE_{T}-WFE_{T-1})/WFE_{T-1}}{(GDP_{T}-GDP_{T-1})/GDP_{T-1}}$$ (5)

In this paper, the decoupling state was divided into eight categories based on the magnitude of the decoupling index in Table 1. Taking the decoupling state of WFE-GDP as an example, the most desirable decoupling state was strong decoupling, where water consumption and economic growth showed an inverse relationship, indicating that economic growth was no longer dependent on the increase in water consumption of energy and that the efficiency of water consumption increased and entered a sustainable use phase. The state of strong negative decoupling was the least desirable, suggesting that economic development had not been enhanced with water consumption of energy.

**Table 1:** Tapio decoupling status system table.

Decoupling state	Type	ΔWFE_t_	ΔTWC_t_ or ΔGDP_t_	e
Strong decoupling	I	<0	>0	e<0
Week decoupling	Ⅱ	>0	>0	0<e<0.8
Recession decoupling	III	<0	<0	e>1.2
Strong negative decoupling	Ⅳ	>0	<0	e<0
Recession connection	Ⅴ	<0	<0	0<e<0.8
Expansion negative decoupling	Ⅵ	>0	>0	e>1.2
Recession connection	Ⅶ	<0	<0	0.8<e<1.2
Expansion connection	Ⅷ	>0	>0	0.8<e<1.2

#### 2.2.3 Lasso model

There are many driving factors of WFE, and omitting significant independent variables or introducing more irrelevant independent variables in the modeling process will reduce the accuracy of model estimation and prediction. Based on this, this paper adopted the Lasso model to identify the driving factors of WFE, to filter out the factors that significantly impact on WFE.

Lasso regression is a kind of shrinkage estimation. By imposing constraints based on OLS, the coefficients of non-significant independent variables are compressed to 0, then influential independent variables are screened, and optimal models are constructed, which can avoid the influence of multicollinearity among variables. Based on the initial selection of driving factors of WFE, reference is made to the adaptive Lasso model constructed by Zou [60] to select the driving factors of WFE. Therefore, this paper firstly standardizes the data related to the drivers and secondly compresses the model regression coefficients using constraints to select effective factors, and the specific adaptive Lasso [61] constructed is as follows:

$$\overset{\wedge }{\beta }=\arg\;\min_{\beta }\sum_{i=1}^{N}{\left(y_{i}-\beta X_{i}\right)}^{2}+\lambda \sum_{q=1}^{z}\omega_{q}\left|\beta_{q}\right|$$ (6)

Where $\omega_{q}=(|\beta_{q.(OLS)}|{)}^{-\gamma },$ *ω*_*q*_ denotes the weights, $\beta_{q.(OLS)}$ is the least squares estimator of *β*_*q*_, *γ* means the adjustable parameters. The optimal regression coefficients $\overset{\wedge }{\beta }$ of the adaptive Lasso model are obtained by combining the expanded Akaike Information Criterion (AIC), Bayesian Information Criterion (BIC), and other criteria in selecting drivers using the adaptive Lasso model. A positive coefficient of indicates that the driving factor has a positive effect on the WFE and, conversely, a negative effect.

In identifying the key driving factors of WFE in Northeast China using the Lasso model, this article follows these steps. Firstly, this paper uses STATA to standardize variables such as WFE, water endowment, economic development, energy consumption, environmental governance and life quality. Secondly, combined with AIC, BIC and EBIC criteria, the adaptive Lasso model is used to regress the standardized variables and identify the key driving factors. Thirdly, OLS regression is performed on the identified driving factors to obtain the estimated results.

### 2.3 Variable design and data sources

#### 2.3.1 WFE calculation

Based on the classification of WFE in the previous section, the PWFE in the energy production process is shown in Table 2 [62, 63]. Due to the complexity of the fossil energy production process, the calculated direct water footprint of fossil energy was used as the WF of fossil energy in this paper.

**Table 2:** PWFE in energy production life cycle in China.

Energy		Production process	Categories	PWFE
**Fossil**	Coal	Mining	PWFE_b,d_	0.010m^3^/GJ
			PWFE_g,d_	0.288m^3^/GJ
		Processing	PWFE_b,d_	0.005m^3^/GJ
			PWFE_g,d_	0.036m^3^/GJ
	Crude oil	Extraction	PWFE_b,d_	0.167m^3^/GJ
			PWFE_g,d_	0.016m^3^/GJ
		Processing	PWFE_b,d_	0.068m^3^/GJ
			PWFE_g,d_	—
	Natural gas	Extraction	PWFE_b,d_	0.077m^3^/GJ
			PWFE_g,d_	0.0195m^3^/GJ
		Purification	PWFE_b,d_	0.006m^3^/GJ
			PWFE_g,d_	—
**Power**	Thermal power	Cooling	PWFE_b,d_	0.681m^3^/GJ
			PWFE_g,d_	0.083m^3^/GJ
		Energy inputs	PWFE_b,in_	0.063m^3^/GJ
			PWFE_g,in_	0.387m^3^/GJ
	Hydropwer	Evaporation & Leakage	PWFE_b,d_	6.75m^3^/GJ
		Dam construction	PWFE_b,in_	0.002m^3^/GJ
	Nuclear power	production	PWFE_b,d_	0.015m^3^/GJ
		Uranium mining & Fuel processing	PWFE_b,in_	0.086m^3^/GJ
	Wind power		PWFE_b,in_	0.0005m^3^/kWh
	Solar power		PWFE_b,in_	0.265m^3^/kWh

#### 2.3.2 Driving factors

Before adopting the adaptive Lasso model to screen driving factors, this paper referred to the existing literature [49, 50] and constructed the indicator system of driving factors from the aspects of water endowment, economic development, energy consumption, environmental governance, and life quality, as shown in Table 3. Energy-intensive industries referred to industries that consumed a high proportion of energy or have a strong dependence on energy, and this paper adopted the delineation standards for high-energy-consuming industry, the manufacturing industry of chemical raw materials and chemicals, the non-metallic mineral products industry, the ferrous metal smelting and calendering industry, the non-ferrous metal smelting and calendering industry, and the production and supply of electric power and heat.

**Table 3:** Indicator system for the driving factors of WFE.

Driving Factor	Indicator	Calculation Method
Water Endowment	TW	total water
	WUR	utilization ratio of water = water availability / total water
	WSTR	water structure = industrial water / total water
Economic Development	PGDP	per capita GDP = GDP / total population
	ECST	economic structure = gross output of energy-intensive industry / GDP
	UR	urban = urban population / total population
Energy Consumption	EPR	energy price = industrial producer ex-factory price indices
	EEF	energy efficiency = GDP / energy consumption
	ENST	energy structure = consumption of coal and crude oil / energy consumption
Environmental Governance	ENR	environment regulation = investment in industrial environmental treatment / value added of the secondary sector
	GIT	green technology invention
Life Quality	PI	actual per capita disposable income
	PE	actual per capita consumption expenditure

#### 2.3.3 Data

The basic data of this paper was adopted from Northeast China from 2005 to 2020. Among them, the fossil energy production data of the region was from the "China Energy Statistics Yearbook" and provincial "statistical yearbooks", the power data was obtained from the China Electricity Yearbook, and the data on the low-level heat generation of energy was from the 2017 "China Energy Statistics Yearbook". The unit of fossil energy production was unified to 10^4^ ton coal equivalent (tce), and the unit of power production was unified to 10^8^ kWh, and the average low calorific value (GJ/10^4^ ton) and PWFE (m^3/^GJ) of all kinds of energies were used to calculate the WFE of energies. Of the data on driving factors, the data on water endowment, economic development, and life quality were from provincial provincial "statistical yearbooks", and GDP, PI, and PE were deflated using 2004 as the base period to reduce the disturbance caused by inflation. The data on energy consumption was from "China Energy Statistics". EPR, GDP in the calculation of EEF were also adjusted to the 2004 base period, and the unit of energy consumption was unified to 10^4^ tce. The data on environmental governance was from "China Environmental Statistics Yearbook" and the National Bureau of Statistics. GIT was the number of green inventions filed by the region that year.

## 3 Result analysis

### 3.1 Calculation and analysis of WFE in Northeast China

#### 3.1.1 Regional analysis of WFE

**Fig 1:**
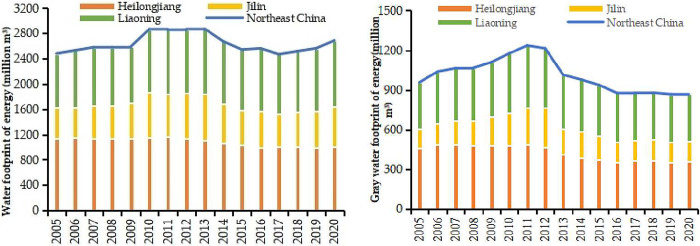
WFE and GWFE in Northeast China from 2005 to 2020.

The changes of WFE in Northeast China from 2005 to 2020 are shown in Fig 1. In terms of total volume, the WFE showed a trend of up-down-up, with an increase of about 1.08 times from 2005 to 2020, while the total water increased by 1.43 times during the same period, which indicated that the development of energy industry is exerting some pressure on water [64]. During the study period, the WF of fossil energy showed an up-down trend, while the WF of power was always on the upward trend. However, the WF of fossil energy decreased while the rising speed of electric energy energy was first small and then large, so that the WFE in Northeast China showed the up-down-up trend.

In terms of composition, the WFE of Heilongjiang and Liaoning were relatively large, with both accounting for more than 80%. The average WFE of Heilongjiang was 1085.87 million m^3^, and its WFE decreased from 1131.31 million m^3^ in 2005 to 1010.96 m^3^ in 2020. The reason was that the WF of fossil energy, which accounted for more than half of the total, continued to decline at a faster rate than power growth. The WFE of Liaoning increased from 860.96 million m^3^ in 2005 to 1040.64 million m^3^ in 2020, showing a trend of up-down-up. This is mainly because the WF of fossil energy showed an up-down trend, and the WF of power continued to rise, and the rising rate was first smaller than and then larger than the decline rate of fossil energy in the same period. The average WFE of Jilin was 588.24 m^3^ from 2005 to 2020, and accounted for the most minor proportion in Northeast China. In Jilin, the WF of fossil energy showed an up-down trend, and the WF of power continued to rise, and the rising speed was smaller than the decreasing speed of fossil energy in the same period, resulting in the overall WFE showing the up-down trend.

Compared with the WFE, the GWFE in various provinces showed a trend of rising first and then decreasing (as shown in Fig 1). The GWFE of the three provinces increased from 957.25 million m^3^ to 1236.27 million m^3^ during 2005–2011 and then decreased to 872.62 million m^3^ in 2020. This also confirmed that water utilization was gradually improving. Jilin was the province with the lowest proportion of the GWFE in Northeast China. The average annual GWFE in Heilongjiang and Liaoning were 425.50 and 394.19 million m^3^ respectively, which was related to the different energy structures in the three provinces.

#### 3.1.2 Category analysis of WFE

**Fig 2:**
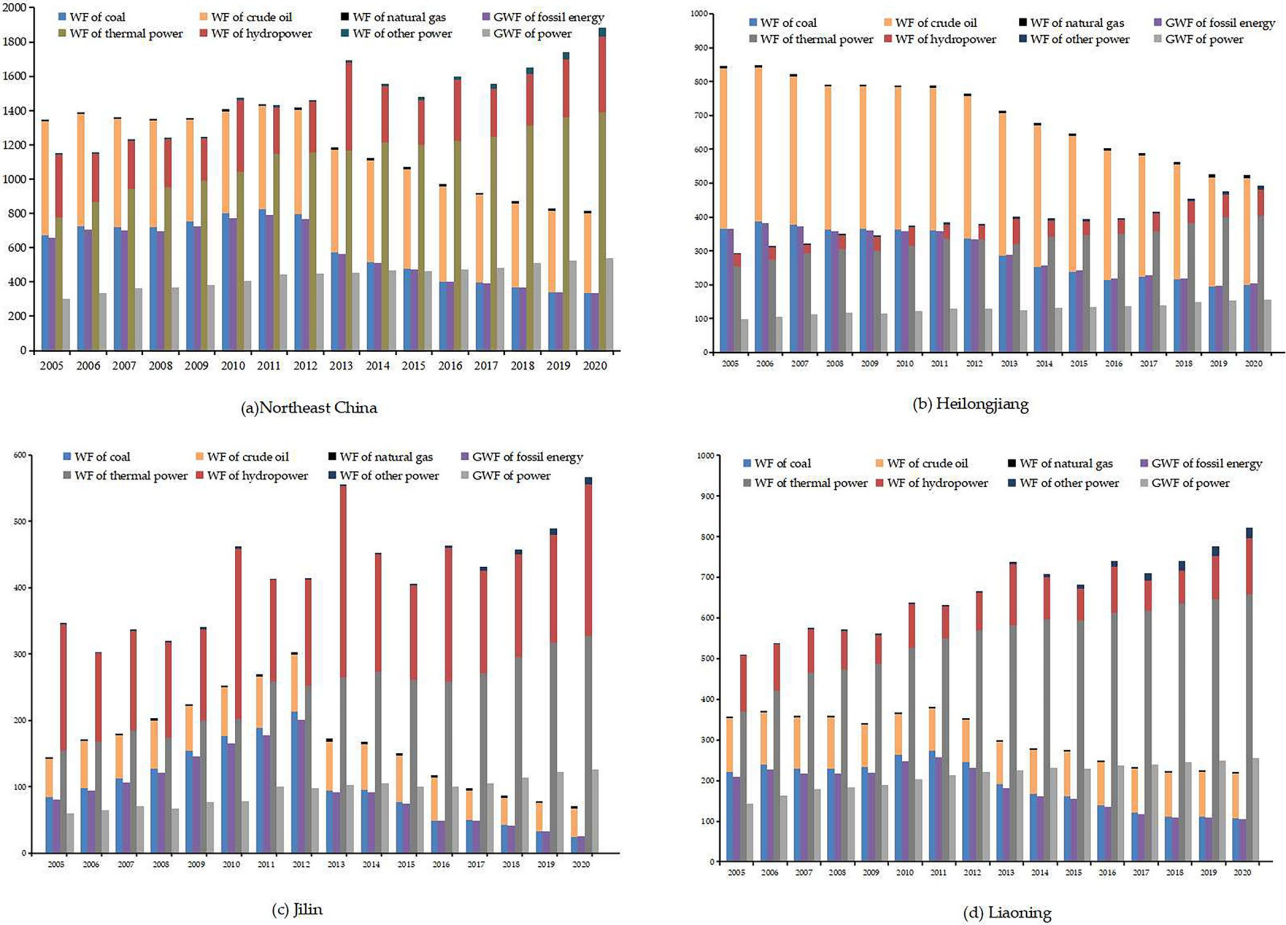
WF of different energies in Northeast China from 2005 to 2020.

According to the evolutionary trend of fossil energy in Northeast China in Fig 2(A), the WF of fossil energy was dominated by coal and crude oil. The WFE of coal and crude oil showed the up-down trend over the study period with 2011 as the cut-off point, with the WF of coal declining the most at 310 million m^3^. The Government of China has incorporated measures such as "the Construction of Ecological Civilization", "Energy Conservation and Emission Reduction Plan" and "Energy Development Planning" into its national plans, so the fossil energy structure has been optimized. The GWFE accounted for 48% of the WFE of fossil energy, and its time-series evolution characteristics were the same as that of fossil energy, reaching the highest value of 792.29 million m^3^ in 2011.

The WF of power showed an upward trend, with a 62% increase in 2020 compared to 2005, mainly because the power industry continued to increase its power production due to national policies. For the structure of WF of power, the WF of thermal power accounted for more than 75% but was decreasing year by year. The WF of hydropower showed a slowly rising trend, accounting for 22.5% of the total, and the proportion of WF of other power was low but showed a continuously rising trend. This is mainly due to the slower development of clean energy technologies in Northeast China. For the GWFE, the time-series evolution characteristics were the same as that of power, with a maximum of 537.86 million m^3^ in 2020. The proportion of GWFE was below 30% and on a decreasing trend.

Comparing the WF of fossil energy with that of power, this paper found that the WF of power was larger than that of fossil energy after 2010, and the gap increased year by year. The ratio of WF of fossil energy to that of power grew from 1:0.85 in 2005 to 1:2.33 in 2020, while the ratio of GWFE grew from 1:0.46 to 1:1.61. That indicated the increase of power production in the energy structure could fully utilize water and reduce water pollution.

The evolutionary characteristics of WF of various energies differed in the three provinces in Fig 2(B)-2(D). The WF of fossil energy in Heilongjiang was larger than that of power during the study period. The production of fossil energy, mainly crude oil and coal, was decreasing due to the reduction in reserves and the increasing difficulty of extraction, leading to a downward trend. As a result, the WF of raw coal and crude oil decreased. While the WF of natural gas was on an upward trend and higher than that of the other two provinces. The WF of power, dominated by thermal power, was on the rise. Among them, non-fossil energy such as hydropower, wind power, and solar power were increasingly favored. Wind power had a huge potential for extraction and required minimal water consumption, with only 0.1 m³of water needed to extract 1MW of wind power.

In Jilin, the WF of fossil energy was lower than that of power, and was also lower than that of the other two provinces. Jilin has a relatively rich variety of energy sources, with relatively small reserves of traditional energies such as coal, oil, and natural gas, while new energies like solar and wind power has significant endowment advantages. During the government’s 13th Five-Year Plan period, Jilin has increased the development and utilization of new types of energies, and renewable energy has fully entered a stage of large-scale development. In the field of power, the WF of new energies such as hydropower, wind power, and solar power was on par with that of thermal power, and the upward trend was evident.

For Liaoning, the WF of power was always higher than that of fossil energy, and was also higher than that of the other two provinces. Liaoning still relied mainly on high water-consuming energies such as coal, oil, and power. The power industry was still primarily based on water-intensive thermal power generation, with the WF of thermal power consistently increasing and higher than that of the other two provinces. The WF of nuclear power, wind power, solar power, and hydropower was growing fast, but there was still a need to step up the pace of construction.

Comparing the GWFE of various energies in the three provinces, this study found that the GWFE in Jilin and Liaoning was smaller due to the relatively larger proportion of power in the energy. The grey water footprint of power in Jilin was smaller because of the relatively high share of hydropower. Therefore, this suggests that an increase in the percentage of hydropower in the energy structure will reduce water pollution and increase water efficiency.

### 3.2 Dynamic evolution of WFE decoupling effects

#### 3.2.1 Analysis of WFE-TWC decoupling effects

**Fig 3:**
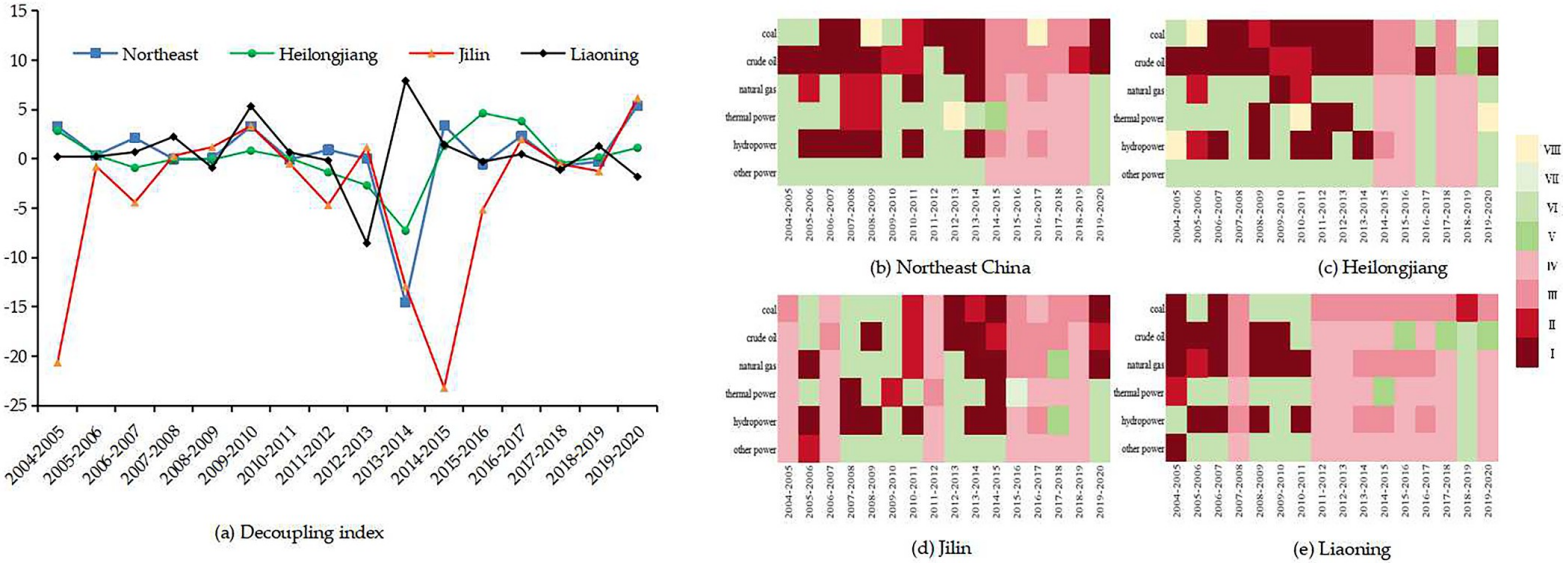
Decoupling effects of the WFE-TWC in Northeast China from 2005 to 2020.

Based on the decoupling model Eq (4)–(5), Fig 3(A) shows the decoupling indexes of WFE-TWC in Northeast China. The decoupling states in Northeast China in 2005–2020 were mainly manifested as strong decoupling, weak decoupling, and strong negative decoupling. The states of decoupling varied among the three provinces. The decoupling status of Heilongjiang was dominated by strong decoupling and weak decoupling. The decoupling states of Jilin and Liaoning showed a strong negative decoupling, which was an undesirable decoupling status, indicating that water consumption of energy put more pressure on water and that they do not develop in harmony with each other.

In Fig 3(B)–3(E), coal-TWC and crude oil-TWC in Northeast China maintained a coordinated state from 2005–2014, but the degree of dissonance intensified in 2014–2020. The decoupling states of coal and crude oil in all provinces except Heilongjiang were unsatisfactory, especially for crude oil, which showed stronger recession decoupling status. In fossil energy, the decoupling status of natural gas was the least favorable, with all provinces showing expansion negative decoupling and recession decoupling status. The decoupling status of natural gas in Liaoning was dominated by strong decoupling in 2005–2011 but worsened after 2011.

None of the three provinces had achieved decoupling status of thermal power, especially Liaoning. The decoupling state of the hydropower had been in disarray since 2011 and was closely related to the policy of accelerating electrification by aggressively constructing hydropower projects in Northeast China. The region’s aggressive energy transition and development and utilization of clean energies, such as wind and solar, had contributed to the increase of water consumption of clean energy and the deterioration of the decoupling relationship.

#### 3.2.2 Analysis of WFE-GDP decoupling effects

**Fig 4:**
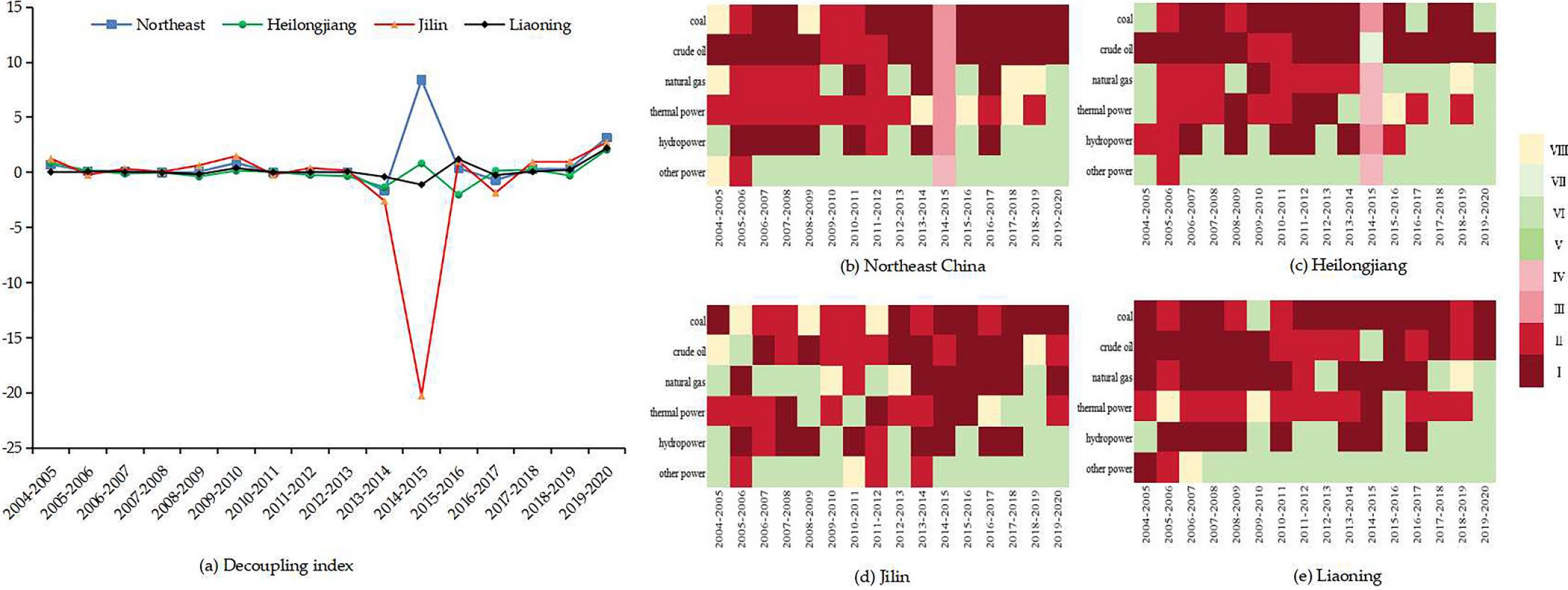
Decoupling effects of the WFE-GDP in Northeast China from 2005 to 2020.

As shown in the Fig 4(A), the decoupling state of WFE-GDP was better than that of WFE-TWC in Northeast China. The decoupling status of WFE-GDP showed strong decoupling and weak decoupling from 2005 to 2020, which reflected that Northeast China was achieving the objective of economic growth while making efficient use of water [65]. Heilongjiang reached the decoupling state. Liaoning’s decoupling indexes were relatively steady, showing the ideal decoupling status. Jilin had the relatively poorer decoupling state, with the fluctuating decoupling indexes, but showed the decoupling status overall.

The decoupling states of WF of various energies from GDP by regions are presented in Fig 4(B)–4(E). In fossil energy, coal and crude oil were the most ideal decoupled energies among the six energies. China’s strategies for regulating the extraction of coal and crude oil were not uncontrolled, so the WFE was not strongly driven by high-speed economic development. For natural gas, nearly half of the decoupling states in Northeast China were in the status of non-decoupling, especially in Heilongjiang. In recent years, the fossil energy transition in Heilongjiang had caused the rapid increase of natural gas production, so the coordinated relationship between the region’s economy and its WFE was undermined.

The decoupling status of power was less favorable than that of fossil energy. The decoupling status of thermal power was dominated by weak decoupling. The decoupling state of hydropower was not as good as that of thermal power, and its decoupling status in the three provinces mainly was expansion negative decoupling status. For other power, we found that the decoupling status of the three provinces was dominated by the expansion of negative decoupling. Other power in Heilongjiang and Jilin was dominated by wind, and other power in Liaoning was dominated by wind and nuclear in recent years.

Overall, the WFE-TWC and WFE-GDP reconciliations are deteriorating, especially power, which is a wake-up call for future water use in energy production. Therefore, it is vital to improve the water efficiency of energy production facilities, improve recycled water recycling and cooling technologies, and achieve improvement in electricity production efficiency through technological change, thereby curbing the growth in water consumption resulting from increased power production.

## 4 Analysis of the driving factors of WFE in Northeast China

### 4.1 Regional analysis of the driving factors of WFE

This paper identified the driving factors of the WFE in Northeast China based on the adaptive Lasso model. To verify the robustness and validity of the adaptive Lasso model regression, the driving factors were subjected to stepwise regression and OLS regression in this paper, and the estimation results of parameters were consistent. The specific results are shown in Table 4.

**Table 4:** Regression results of the adaptive Lasso, stepwise regression and OLS for the driving factors.

Variables	Northeast China			Heilongjiang	Jilin	Liaoning
	Adaptive Lasso	Stepwise regression	OLS	Adaptive Lasso		
	Coefficient	Coefficient	Coefficient	Coefficient	Coefficient	Coefficient
TW	0.5914^***^	0.5501^***^	0.6450^***^		0.3659^***^	
WUR	0.2923^***^	0.2990^***^	0.2940^***^			-0.0990^**^
WSTR			-0.0996	0.0465	0.2424^***^	
PGDP	0.3744^**^	0.3333^**^	0.4468^**^	0.1978^***^	0.5325^***^	0.0996
ECST			-0.1413	0.3489^***^		0.1262
UR	1.1844^***^	1.3889^**^	0.8338	-0.2056^***^		
EPR	0.0685		0.0780			
EEF	0.6721^***^	0.7076^***^	0.7100^***^			-0.2256^***^
ENST	0.9050^***^	0.9295^***^	0.8563^***^			
ENR	-0.1465^*^	-0.1856^**^	-0.1123			
GIT	0.1571		0.0207			
PI	-0.2955	-0.3307^*^	-0.9850^*^			
PE			1.0974			
*F Test*	34.87	26.41	27.24	117.92	155.11	15.44
*R-squared*	0.8507	0.8122	0.8604	0.9710	0.8652	0.6951

Notes

***p < 0.01,

**p <0.05,

*p < 0.1.

The variables that had large and significant driving effects on the WFE of Northeast China included TW, WUR, PGDP, UR, EEF, ENST, and ENR in Table 4. The influence of UR played the most significant role. With the implementation of the strategy of revitalizing the old industrial bases, Northeast China has become a developing region with rapid industrialization and urbanization, which is accompanied by a large amount of energy production and consumption [66]. The economic development of Northeast China is still dependent on energy, and the industrial structures of most cities are still dominated by heavy chemical and resource-intensive industries [67, 68], whose energy consumption is dominated by low extraction cost and highly polluting coal. In addition, ENR had the dampening effect on WFE. So improving water efficiency for fossil energy (mainly coal) [31] and formulating policies and measures to constrain energy and water use [69] are crucial to alleviate the energy-related water nexus.

The results of identifying the driving factors of WFE and parameter estimation for the three regions in Northeast China are shown in Table 4. PGDP and ENST in Heilongjiang significantly drove the growth of WFE. Heilongjiang was an important old industrial base with rich coal and oil, higher demand for fossil energy in heavy industry and agricultural, and still higher water abstraction intensity for fossil energy extraction and thermal power [39]. In Jilin, TW, WSTR, and PGDP showed significant positive driving effects on WFE. The proportion of industrial water to total water was the highest in Jilin in the study interval, about 17.6%, and showing a gradual downward trend, which suggested that a decline in the industrial water consumption would inhibit the growth of Jilin’s WFE. For Liaoning, WUR and EEF had dampening effects on WFE instead. Due to the optimized water structure, the faster development and utilization of less water-intensive clean energies, which in turn reduces the water consumption. The above analyses showed that there were differences in the driving factors and effects of WFE among regions in Northeast China.

### 4.2 Category analysis of the driving factors of WFE

#### 4.2.1 Analysis of Northeast China

The identifications of driving factors of WF of different energies in Northeast China are shown in Table 5.

**Table 5:** Identification results of driving factors of different energies in Northeast China.

Variables	Fossil energy			Power		
	Coal	Crude oil	Natural gas	Thermal power	hydropower	Other power
TW	0.5634^***^	0.4999^***^	0.6625^***^	0.1116^**^	-0.1121	
WUR	0.3407^***^	0.3039^***^	0.2871^***^	0.1094^***^	-0.4345^***^	-0.0612
WSTR		-0.0700	-0.1233		0.2735	0.2568^***^
PGDP	0.2914			0.3443^***^	0.2633	
ECST		-0.1690^*^	0.1667^**^	-0.0464	0.1331	-0.4407^***^
UR	0.6391^*^	0.8908^**^		0.8490^***^	-1.3540^***^	0.5161^**^
EPR	0.0950	0.0435	0.1006^*^	0.0252	-0.0233	0.0773^*^
EEF	0.4574^***^	0.7745^***^	0.2443^***^	0.0802^**^	-0.4467^***^	
ENST	0.6259^**^	0.9894^***^	0.4280^***^	0.1525^*^	-0.6609^**^	-0.1728^**^
ENR	-0.2047^**^	-0.1100^**^	0.0653	-0.0544	0.2908^***^	
GIT	0.1364	0.1502	-0.0644	0.0367	-0.2163	
PI	-0.6755^***^		1.6686^***^	-0.7216^***^	0.8153^**^	
PE			-1.2315^**^	0.5901^**^		0.6488^***^
*F Test*	26.87	45.10	52.38	492.67	13.84	46.03
*R-squared*	0.8538	0.9119	0.9299	0.9883	0.8154	0.9349

Notes

***p < 0.01,

**p <0.05,

*p < 0.1.

For fossil energy, the indicators of water endowment and energy consumption had significant positive driving effects, and ENR had the inhibiting impact. Strict environmental constraints stimulated the development of renewable energy and the transition to clean energy [70], inhibiting the rise of WF of crude oil and crude coal. However, PE had the negative impact on the WFE of natural gas, which suggested that the demand for natural gas was low on the consumption side. Despite natural gas in Northeast China can only meet regional self-sufficiency, it is gradually applied in the production and living areas. But compared with traditional fossil energies, natural gas is more expensive so that the energy at the consumption end (e.g., heating) is still dominated by coal, which is not conducive to alleviating the pressure on water [71].

In terms of power, there were also differences in the driving factors and effects of WFE between power in Northeast China in Table 5. The significant driving factors of WF of power were the indicators of water endowment shown in Table 5. The positive driving effect of PE indicated the predominance of thermal power on the consumption side, while the negative driving effect of PI suggested the optimization of the energy mix transition on the production side in Northeast China. Positive driving factors of the WF of hydropower were significantly inhibited by variables other than PI and ENR. Despite the 33.3% water development and utilization rate in Northeast China, hydropower accounted for only 2.5% of the total power in 2023 because hydropower was the water-intensive energy and there were constraints on its utilization such as a weak grid structure [68]. Other power production accounted for a relatively small proportion but was worth paying attention to. Northeast China had abundant renewable and clean energies, such as wind power, hydropower, geothermal energy and nuclear energy. Increasing the penetration rate of renewable energies with low-pollution and low-water-consumption could achieve synergistic effects of water conservation and emission reduction [18]. Therefore, PI and PE have a greater impact on the WF of power in Northeast China.

#### 4.2.2 Analysis of the three regions in Northeast Chin

**Fig 5:**
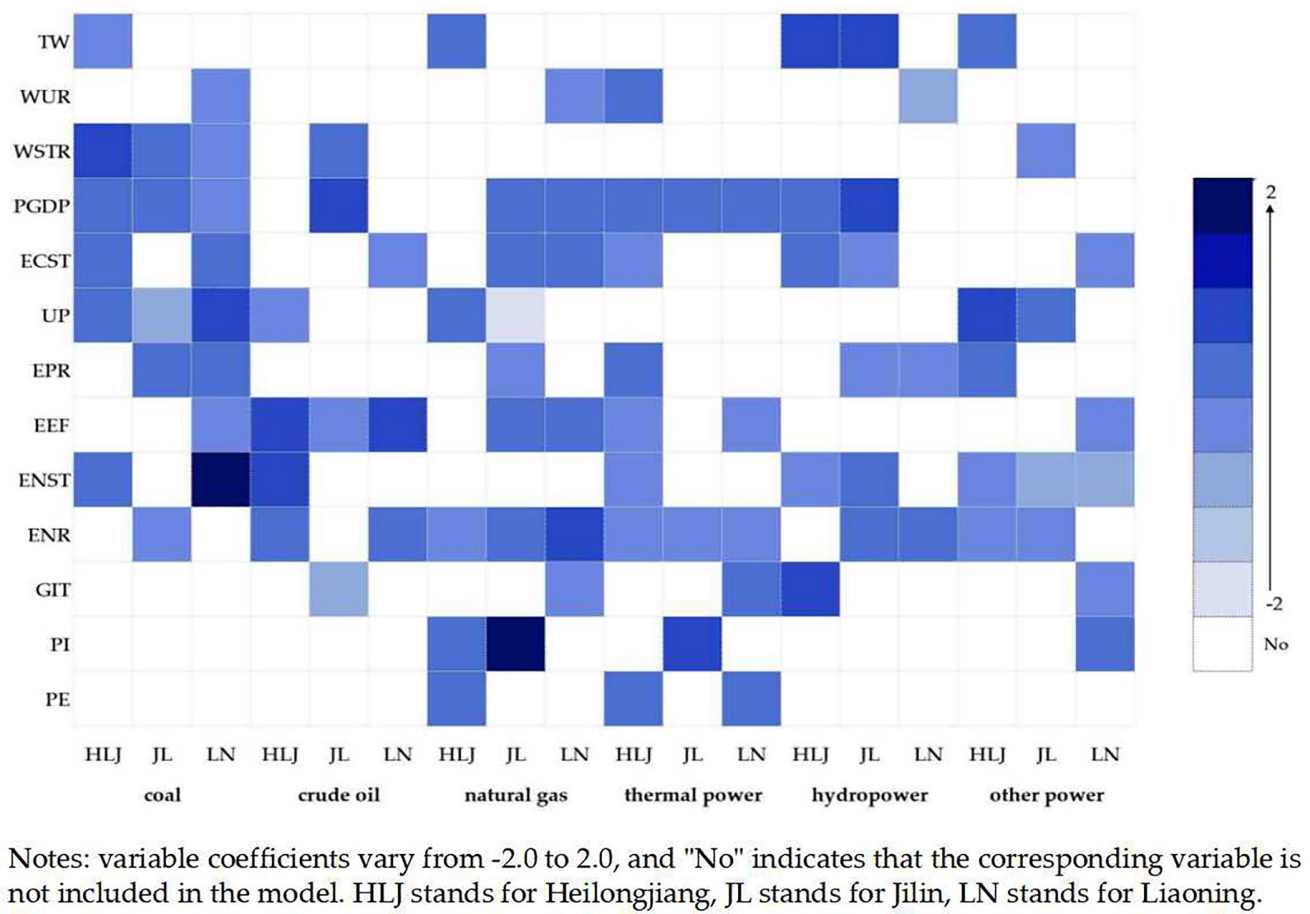
Identification results of driving factors of different energies in three regions.

The identifications of the driving factors of WF of different energies in the three regions of Northeast China are shown in Fig 5.

Based on the driving factors of WF of fossil energy by provinces, ECST and ENST played incremental roles in the WF of coal and crude oil relatively. Relying on the Daqing oilfield, which has a cumulative production of 2.5 billion tonnes, the abundant oil resources not only meet the industrial needs of Heilongjiang but also transfer a large amount of oil out of the province, increasing the amount of water consumed. ENR had the significant dampening effect on the WF of coal but the significant driving effect on the WF of natural gas in Jilin, implying that investment in environmental pollution control promoted the production of natural gas, a cleaner energy source, and was conducive to internal optimization of the fossil energy structure. For Liaoning, the indicators of economic development drove the WF of coal and natural gas, while the industrial-based economic structure inhibited the growth of the WF of crude oil. This is due to the structural reform of the petrochemical industry side, and vigorously develop the refined chemical industry. The above analysis shows that the indicators of economic development, energy consumption structure and environmental regulation play different roles in the WF of fossil energy in the three regions.

The driving factors of WF of power in different provinces are heterogeneous. PE played the incremental role in the WF of thermal power in Heilongjiang. The improvement in people’s life quality makes the higher demand for electricity, which is bound to increase the consumption of electricity dominated by thermal power. However, the backwardness of thermal power generation technology in Heilongjiang consumed more coal and water to produce the same amount of electricity [72]. Economic development indicators in Jilin significantly drove the growth of WF of thermal power and hydropower which also explained Jilin’s comprehensive construction of a new power system and the creation of a "green power" industry to achieve a clean energy transition. ECST and ENST inhibited the WF of other power in Liaoning, which was related to the strategy of eliminating outdated production capacity and restructuring of the traditional energy industry. Liaoning proposed to cultivate and grow new energy industries such as wind power, promote the safe and efficient use of clean and low-carbon energy, and adjust the structure of energy consumption. Therefore, the effects of economic development indicators, energy consumption structure and consumption expenditure on the WF of power in the three regions are different.

## 5 Conclusions and policy implications

### 5.1 Conclusions

Based on the ISO model, this paper analyzed the dynamic evolution of WFE in Northeast China, measured the decoupling effects of WFE-TWC and WFE-GDP using the Tapio model, and identified the driving factors of WFE in Northeast China based on the Lasso model, and obtained the following conclusions:

First, the WFE of Northeast China showed a trend of up-down-up. There were differences in the evolution of WFE in regions and categories. Heilongjiang had the highest WF of fossil energy and natural gas. The WF of power increased, and wind power had a huge potential for extraction and required minimal water consumption, with only 0.1 m³of water needed to extract 1MW of wind power. Jilin had the smallest WFE, with a relatively high proportion of the WF of power. The utilization level of new energies continued to rise, with wind power utilization nearly 98%, and photovoltaic utilization nearly 99%. The WFE of Liaoning was primarily dominated by thermal power. The province suffered from a scarcity of fossil energy and limited development space for clean energy. Its energy consumption was coal-heavy, and there was an urgent need to improve its power supply capacities.

Second, the decoupling status of WFE-GDP was better than that of WFE-TWC in Northeast China. All three regions showed a decoupling status for WFE-GDP, but there were differences in categories. The decoupling status of natural gas-GDP in Heilongjiang was not ideal. Due to the rapid increase in natural gas production in Heilongjiang, from 324.9 tce in 2005 to 622.2 tce in 2020. Hydropower and other power had not decoupled from GDP and TWC. Affected by the financial crisis, the economic growth rate of the three provinces had slowed down, but the development of hydropower had been accelerating, leading to the disruption of the coordinated relationship between regional economic development and electricity water consumption. Taking Jilin as an example, the installed capacity of hydropower increased to 5.1 million KW in 2020, which was closely related to the active construction of small hydropower projects and the policy of accelerating rural electrification.

Third, the driving factors of WFE were heterogeneous between regions and energies. The effects of indicators of water endowment, economic development, and energy consumption were significant in Northeast China, with economic development indicators having more significant positive driving effects in Heilongjiang and Jilin, primarily related to the high-energy-consuming, heavy industry economic structure of the regions. The indicators of economic development and life quality showed significant driving effects in energies. Environmental regulation inhibited the WF of coal and crude oil, and life quality indicators strongly drove effect on the WF of power.

### 5.2 Policy implications

This paper provided a complementary perspective on the WFE of Northeast China. Northeast China should take into account the actual situations and the effects of driving factors, define the governance priorities for the coordinated development of energy and water, and improve and formulate measures and policies to reduce energy and water consumption by adhering to the governance methods of "regional specialization and regional co-ordination" and "by region and by category".

(i) Strengthen regional specialization and coordinated development. Firstly, areas with high dependence on fossil energy should upgrade technology to reduce the water consumption at all stages of fossil energy development and utilization. Secondly, areas with high power consumption could transform the internal structure to reduce water use and air pollution generated by thermal power. Thirdly, as the energy-water constraint region in China, Northeast China should rationally adjust industrial structures, increase the proportion of clean energies such as natural gas that had lower WF per unit of energy output, develop the energy industry reasonably based on resource endowment, and promote the coordinated development of water and economy through the transformation of energy utilization methods.

(ii) Precise policies vary from regions and categories. The region could be combined with the actual situation and the different driving factors of WFE, and clear priorities for regional energy and water governance.

Firstly, as the region with the highest consumption of fossil energy, Heilongjiang should accelerate the low-carbonization and electrification of energy use, deeply implement the "Gasification of Heilongjiang" project, strictly control the coal-fired power installations. In the meantime, the region could also accelerate the substitution of renewable energy, introduce clean energies such as solar and wind energy, develop new types of energy storage, and accelerate the implementation of clean energy heating to add new drivers for economic growth. Furthermore, Heilongjiang can strengthen policy supervision in key areas, encourage industrial and agricultural enterprises to improve the efficiency of energy and water utilization through technology innovation, actively guides local residents’ preferences towards clean energy consumption, and raises awareness of water conservation and energy efficiency.

Secondly, Jilin should optimize its energy consumption structure, develop the high-quality wind energy, solar energy, biomass energy, and other resources, and accelerate the growth of non-fossil energy power generation, coordinating the supply of various energy sources such as oil, gas, and electricity. The region should also implement tiered electricity and gas pricing policies, and pay attention to the water resource efficiency of different electric power technologies [73]. Furthermore, Jilin should also actively implement corporate tax policies that support energy conservation, strengthen environmental pollution control, and promote energy-saving technologies in the industrial sector to achieve water resource, conservationthereby increasing water and energy security, improving water quality and air quality, and positively enhancing the quality of life and health levels of local populations.

Finally, Liaoning can orderly construct a green, efficient, and stable diversified electricity supply pattern. This includes the reasonable arrangement of thermal power peak shaving projects, the safe and steady development of nuclear power, the systematic advancement of pumped storage power station construction, the active promotion of inter-provincial ultra-high capacity power transmission corridor construction, and gradually forming an integrated internal and external grid system of Liaoning with the Northeast and North China regional grids. At the same time, Liaoning can improve the water resource efficiency of energy generation through policy management, upgrade the water resource utilization technology of hydropower, strengthen the proportion of non-thermal power generation on the consumption side, and optimize the energy driving factors in economic growth. This can promote green and sustainable economic development, drive the development of related industries, and increase employment opportunities for local residents.

However, this study provides preliminary evidence on the water consumption of fossil energy and power in Northeast China, but some limitations need further exploration. Firstly, this paper calculates the direct water footprint of fossil energy as the WF of fossil energy, ignoring the indirect WF of fossil energy. In the future, with the improvement and refinement of statistical data, the calculation of the WF of fossil energy will become more accurate, and it will also be possible to analyze the role of indirect WF within the WF. Secondly, the clean power calculated in this paper includes solar, wind, and nuclear power. Still energies such as biomass are not considered, which can be further explored in future studies. Finally, the driving factors of energy water footprints are yet to be further refined.

## Supporting information

S1 Data WFE and GWFE of regions and energies. (XLSX)

S2 Data Decoupling effects of the WFE-TWC and WFE-GDP. (XLSX)

S3 Data Driving factors of WFE regions and energies. (XLSX)
